# Associations between Lifestyle Behaviors and Quality of Life Differ Based on Multiple Sclerosis Phenotype

**DOI:** 10.3390/jpm11111218

**Published:** 2021-11-17

**Authors:** Nupur Nag, Maggie Yu, George A. Jelinek, Steve Simpson-Yap, Sandra L. Neate, Hollie K. Schmidt

**Affiliations:** 1 Neuroepidemiology Unit, Centre of Epidemiology and Biostatistics, Melbourne School of Population and Global Health, The University of Melbourne, Parkville, VIC 3010, Australia; maggie.yu@unimelb.edu.au (M.Y.); g.jelinek@unimelb.edu.au (G.A.J.); steve.simpsonyap@unimelb.edu.au (S.S.-Y.); sandra.neate@unimelb.edu.au (S.L.N.); 2 Menzies Institute for Medical Research, University of Tasmania, Hobart, TAS 7005, Australia; 3 Accelerated Cure Project for Multiple Sclerosis, Waltham, MA 02451, USA; hollie@acceleratedcure.org

**Keywords:** multiple sclerosis, lifestyle behavior, MS management, MS phenotype, quality of life

## Abstract

Multiple sclerosis (MS), a neuroinflammatory disorder, occurs as non-progressive or progressive phenotypes; both forms present with diverse symptoms that may reduce quality of life (QoL). Adherence to healthy lifestyle behaviors has been associated with higher QoL in people with MS; whether these associations differ based on MS phenotype is unknown. Cross-sectional self-reported observational data from 1108 iConquerMS participants were analysed. Associations between lifestyle behaviors and QoL were assessed by linear regression, and phenotype differences via moderation analyses. Diet, wellness, and physical activity, but not vitamin D or omega-3 supplement use, were associated with QoL. Specifically, certain diet types were negatively associated with QoL in relapsing-remitting MS (RRMS), and positively associated in progressive MS (ProgMS). Participation in wellness activities had mixed associations with QoL in RRMS but was not associated in ProgMS. Physical activity was positively associated with QoL in RRMS and ProgMS. Phenotype differences were observed in diet and wellness with physical QoL, and physical activity with most QoL subdomains. Our findings show lifestyle behaviors are associated with QoL and appear to differ based on MS phenotype. Future studies assessing timing, duration, and adherence of adopting lifestyle behaviors may better inform their role in MS management.

## 1. Introduction

Multiple sclerosis (MS), a chronic neuroinflammatory disorder, is commonly diagnosed in adults, predominantly women, aged 20 to 30 years [1]. On initial diagnosis, 85% of people with MS (pwMS) are diagnosed with relapsing-remitting MS (RRMS) presenting with acute attacks of new or increasing neurologic symptoms, and 10–15% with primary progressive MS (PPMS) defined by deterioration of symptoms from onset without obvious relapses or remission [2]. Within 15–20 years of diagnosis, approximately 50–75% of RRMS cases convert to secondary progressive MS (SPMS) defined by gradual worsening of neurologic function alongside a general cessation of relapses [3].

Both RRMS and progressive MS (ProgMS) may manifest an array of physiological, psychological, and motor symptoms; the number and severity of these symptoms and associated impairment play a critical role in quality of life (QoL). Symptoms of fatigue, pain, cognitive impairment, depression, and disability are key predictors of worse QoL up to 10 years later [4]. Improvement of symptoms through adoption of healthy lifestyle behaviors has potential to improve QoL.

Healthy lifestyle behaviors, including diet, vitamin D and omega 3 supplementation, and participation in wellness and physical activities have previously been found to be associated with higher QoL. PwMS who adhered to either high quality, MS-specific, or anti-inflammatory diets, have reported improved mental and physical QoL [5,6,7]. Vitamin D supplementation improved QoL in pwMS with initial levels lower than 30 ng/mL [8] and was associated with improved physical QoL in pwMS reporting an average daily intake of over 5000 IU [9]. Less is known about the effects of omega-3 supplement use, though in an international cohort of over 2500 pwMS, those self-reporting frequent fish consumption and taking omega-3 supplements had better QoL [10]. Research on wellness activity participation, ranging from Tai Chi and exercise therapy to mindfulness, relaxation, and imagery has mixed evidence of associations with QoL, though participation is generally reported to be beneficial for physical and mental QoL [11,12,13,14]. The benefits of physical activity for wellbeing are well established, with primarily aerobic forms benefiting social, physical, and mental QoL in pwMS [15].

Though the benefits of healthy lifestyle behaviors on QoL are evident, whether the effects are similar across MS phenotypes is unclear as most studies report on populations of mixed phenotype. As people with ProgMS are generally less responsive to therapies, have greater disability and more severe symptoms than those with RRMS [16,17,18], it is probable that the effects of lifestyle behaviors on QoL also differ. Therefore, we aim to differentiate associations of lifestyle behaviors with QoL between phenotypes, which may provide insight into personalised management strategies specific to disease course.

## 2. Materials and Methods

### 2.1. Study Design and Participants

Commencing from 2014, recruitment to the iConquerMS observational study has been ongoing and open to pwMS and the general population aged ≥21 years. The study is promoted by the sponsoring organization, Accelerated Cure Project for MS, and partner organizations and individuals via online, print and in person communication. Consenting participants are requested to voluntarily complete a series of self-reported online surveys capturing demographics, health and clinical outcomes, as well as lifestyle behaviors, at 6 month intervals. Response to questions at any timepoint is optional.

De-identified baseline data from participants who registered in the study from November 2014 to July 2020 (*n* = 3374) was extracted. Inclusion criteria were participants reporting a clinician-confirmed MS diagnosis, confidence in MS diagnosis, and having completed diet and wellness, physical activity, QoL and disability surveys. RRMS, SPMS or PPMS phenotypes were included, and SPMS/PPMS consolidated. Clinically isolated syndrome, radiologically isolated syndrome, and not sure/don’t know MS phenotype, were excluded. Ethics approval ID #1956113.1.

### 2.2. Demographics and Clinical Outcomes

Age (from date of birth), sex (male, female), highest level of education (no formal education, elementary-middle school, high school, high school graduate, some college, associate degree, technical degree, bachelor’s degree, master’s degree, doctoral degree), partner status (never married, married, divorced, separated, widowed, cohabitation/domestic partner, prefer not to answer), employment status (employed outside home, employed at home, homemaker, student, worker’s compensation, unemployed looking for work, disabled), country of birth (global country list), ethnicity (American Indian/Alaska Native, Middle Eastern, South Asian, other Asian, Black/African American, Native Hawaiian/Pacific Islander, White, don’t know), and annual household income (<USD15,000 to >USD200,001 in increments of USD15,000) were queried and re-categorized.

MS duration was calculated by year of diagnosis and survey completion. Body mass index (BMI) was calculated by weight (kg)/height (m)^2^ then categorized into underweight, normal, overweight, and obese according to World Health Organisation classifications [19]; underweight and normal were combined due to small sample size in the former group. Disability was measured via the Patient Determined Disease Steps (PDDS), and scores collapsed to low (0–2), moderate (3–5) and high (6–8) disability as per guidelines [20].

### 2.3. Lifestyle Behaviors

Variables within diet (*n* = 23), wellness (*n* = 25), vitamins (*n* = 15) and supplements (*n* = 29) categories were each queried via tick-box options of “used” and/or “used and helpful” in the past 6 months to improve health and wellbeing; those not selecting either were considered not using. Two response options were combined for analysis (Yes = used/used and helpful vs. No = none selected). Variables were recategorized for diet and wellness (Table 1) then analysed as a binary variable (Yes = use/used and helpful of ≥1 option within category). Of the vitamins and supplements, only vitamin D and omega-3 were analysed.

Physical activity was assessed via the Godin-Shephard Leisure-Time Physical Activity Questionnaire (GLTPAQ), which queries frequency (0–7 days) of strenuous, moderate, and mild exercise for ≥ 15 min in the preceding seven days [21]. Total leisure activity score was calculated as per guidelines and categorized into sedentary (<14), moderately active (14–23), and active (≥24).

### 2.4. Outcome Measure

QoL was queried via the NeuroQoL Adult Short Form, comprising 13 subdomains, classified under physical, mental, and social QoL [22]. Each of 13 subdomains comprise between five to nine questions scored on a Likert scale. Scores were summated and converted to T-scores (Mean = 50, SD = 10) as per guidelines. For mobility, fine motor, anxiety, depression, positive affect, cognitive function, social participation, and social satisfaction subdomains, T-scores were derived from an average U.S. general population; and for fatigue, sleep disturbance, emotional dyscontrol, and stigma subdomains, T-scores were derived from an average population with a diagnosed neurological disorder (MS, epilepsy, stroke, amyotrophic lateral sclerosis, or Parkinson’s disease). Higher T-scores equate to higher measured concept. T-scores for the communication subdomain were unavailable, therefore raw total score for this subdomain was used for analysis and reporting.

### 2.5. Statistical Analysis

All analyses were conducted in Stata version 15.0 (StataCorp. 2017. Stata Statistical Software: Release 15. College Station, TX, USA: StataCorp LLC.). Associations between lifestyle behavior categories and QoL domain were assessed by multiple linear regression models, adjusted for age, sex, education, BMI, disability, and duration since MS diagnosis, estimating adjusted regression coefficients and 95% CI. An interaction term between MS phenotype and each lifestyle behavior was added to the regression model to assess differences between RRMS and ProgMS.

## 3. Results

### 3.1. Participant Characteristics Based on Phenotype

Of 3374 participants enrolled into iConquerMS, *n* = 1108 (33%) met the inclusion criteria. In the included population, compared to participants with RRMS, people with ProgMS were older, and more likely to be male, not in paid employment, with moderate or severe disability, and longer MS duration (Table 2A). For lifestyle behaviors, compared to RRMS, people with ProgMS were less likely to have used an anti-inflammatory diet, and less likely to be at an active level of physical activity.

For QoL, compared to the clinical for U.S. general population, pwMS reported similar T-score difference (<0.5 SD) in 9 of 13 QoL subdomains, excepting fine motor, mobility, and both social participation and satisfaction (Table 2B), in which people with ProgMS reported marginally lower T-scores. Compared to RRMS, people with ProgMS reported significantly worse QoL in 7 of 13 subdomains: lower mobility, fine motor, positive affect, and both social participation and satisfaction, and higher fatigue and stigma. Anxiety was higher in RRMS (Table 2B).

### 3.2. Associations between Lifestyle and Quality of Life Subdomains

Diet was associated with physical and mental, but not social, QoL (Table 3A). In people with RRMS, anti-inflammatory, low-carbohydrate and other diets were positively associated with stigma, and other diets additionally associated with lower fine motor and cognitive function. In ProgMS, anti-inflammatory diets were associated with higher mobility and positive affect; low-carbohydrate diet with higher positive affect; low-saturated fat diet with higher ease of communication; and other diet with higher mobility. Phenotype differences were observed in mobility and communication subdomains.

Wellness activities were associated with physical, mental, and social QoL (Table 3B). In RRMS, mind activities were associated with lower fine motor, cognitive function, communication, social participation, and social satisfaction, and with higher fatigue, anxiety, emotional dyscontrol, and stigma. Mind-body activities were associated with higher positive affect and social participation, and lower emotional dyscontrol. Other wellness activities were associated with lower physical, mental, and social QoL in 10 of 13 subdomains, excepting mobility, depression, and positive affect. No significant associations were observed between wellness activities and QoL in ProgMS. Phenotype differences were only observed between other wellness activities and the fine motor subdomain.

Physical activity was associated with physical, mental, and social QoL (Table 3C). In RRMS, physical activity was dose-dependently associated with higher mobility, positive affect, and social satisfaction; and with lower anxiety, depression, and stigma. Active level of physical activity was additionally associated with higher fine motor, cognitive function, communication, social participation, and lower fatigue, sleep disturbance, and emotional dyscontrol. In ProgMS, moderate physical activity was associated with higher positive affect, cognitive function, and lower communication; and active physical activity with higher mobility and lower fatigue. Phenotype differences were observed in 8 of 13 QoL subdomains.

Neither vitamin D nor omega-3 supplements use were associated with QoL (Table 3C).

## 4. Discussion

Lifestyle behaviors are known to be associated with QoL in pwMS. To inform potential lifestyle management strategies based on disease course, we assessed associations between diet, vitamin D and omega 3 supplementation, and participation in wellness and physical activities on QoL in pwMS, and whether these associations differed in nature and magnitude between MS phenotypes.

Compared to RRMS, people with ProgMS were older, less likely to be in paid employment, had longer disease duration and greater disability, and had a lower female/male ratio, consistent with previous reports [16,17]. Of lifestyle behaviors assessed, physical activity and QoL differed by phenotype. People with ProgMS were less physically active and had lower QoL in specific physical, mental, and social QoL subdomains, also consistent with prior studies [18,23], and expected given advanced disease stage and greater severity of symptoms adversely affecting QoL, and being likely barriers to performing daily activities and independent living.

High quality, anti-inflammatory, and MS-specific diets have been associated with better mental and physical QoL [5,6]. Our results were mixed and not always aligned with previously reported findings. We identified associations of four diet categories with mental and physical, but not social QoL domains. In RRMS, three of four diet categories were associated with higher stigma, a measure of perceived prejudice and discrimination because of disease, potentially reflective of people who feel greater stigmatisation being more inclined to make changes in their diet in attempt to improve or moderate their condition, or the stigma of adhering to dietary restrictions. Unexpectedly, no positive associations of diet with QoL in RRMS were found; the other diet category, comprising organic, low sodium/sugar and semi-vegetarian diets, was associated with both lower cognitive function and fine motor subdomain scores. Timing of adoption as well as duration and adherence of dietary modification may account for these observations.

In ProgMS, diet was associated with positive affect and ease of communication, perhaps indicative of higher mastery and self-control over MS management. Both anti-inflammatory and other diets were associated with improved mobility, consistent with proposed neuroinflammatory and microbiota-gut-brain-axis disease mechanisms. Though studies have reported associations between diet quality and MS-specific diets with lower depression and fatigue respectively [24,25], we did not observe associations in these symptom subdomains. Discrepancies may be attributable to outcome measure tools in addition to potential additive benefits of adhering to multiple lifestyle behaviors. Phenotype differences were evident only in mobility and communication subdomains. The positive association with mobility in ProgMS, an indicator of disease progression and key contributor to reduced QoL, suggests duration of dietary modification may be a factor, although our data do not allow us to make this conclusion.

No associations between vitamin D or omega-3 supplementation and QoL were observed. Prior studies report mixed evidence for a role of vitamin D supplementation on QoL, with positive associations apparent in pwMS with deficiencies or with an intake of more than 5000 IU/day in addition to sufficient sun-exposure [8,9,26]. Similarly, discrepancies between our observations and that reported for omega-3 and QoL [10], may reflect dose and source of omega-3, or dietary balance of omega-3 and -6. Baseline vitamin and mineral levels, or daily dose, frequency, and duration of supplement use were not captured in the current study.

Participation in wellness activities was associated with physical, mental, and social QoL only for people with RRMS. Mind-body activities, encompassing yoga, Tai Chi, Qigong, and exercise therapy, were associated with positive affect, emotional dyscontrol, and social participation, consistent with past reports of favourable effects of exercise therapy and Tai Chi on mental QoL [12,14]. The non-significant and negative associations observed with mind and other wellness activities with QoL subdomains, some contrary to previously reported [27], may be attributable to category inclusions, adherence to behavior, and/or non-specific symptom assessment. Alternatively, it may be that interactive group wellness activities having positive social interactions may be better interventions for improved mental and social QoL. Phenotype difference was only observed with other wellness and fine motor subdomains. Further investigation capturing information regarding adherence to lifestyle behaviors may provide better insight and is necessary to inform practice recommendations.

The benefits of physical activity on overall health are established [15,28] and supported by our data. We found dose-dependent associations in mobility, social satisfaction, and four mental subdomains in RRMS. Active levels of physical activity were positively associated across 13 QoL subdomains. That common symptoms of fatigue, mobility, anxiety, depression, and cognitive function also showed significant positive associations, highlights the potential value of incorporating regular physical activity in MS management. In people with ProgMS, moderate activity had positive associations for positive affect and cognitive function, and active levels for mobility and fatigue, also encouraging for symptom management through adoption of physical activity. The magnitude of associations was generally stronger in RRMS, especially in active levels. Significant phenotype differences were noted in fine motor, five of seven mental and both social subdomains, suggesting that physical interventions may be best implemented early in disease course, adapted to disease progression.

The strengths of our study are the inclusion of a large and diverse population of pwMS, with minimal participant bias due to the open nature of recruitment, enabling generalizability of findings. Moreover, the large number of participants of RRMS and ProgMS phenotype meant that separation based on disease stage was possible; most prior studies report on mixed phenotype populations. The dataset captures a breadth of clinical and lifestyle variables, enabling robust analysis of associations among a spectrum of behaviors and QoL.

Limitations include self-reported optional survey responses which impact data quality and missingness, and potential selection bias with only 35% participant inclusion for which we controlled by assessing biases between included and excluded participants and adjusting for variables that were significantly different (data not shown). Some participant biases, such as possible increased motivation of pwMS who completed all surveys, are unable to be adjusted for. The cross-sectional analysis limits the inference of causal relationships but provides insight to guide future longitudinal studies. Other factors including socioeconomics, access to health services, and support networks, may also contribute to QoL and should be considered in interpretation of the findings. The use of non-validated tools to capture lifestyle and health outcome variables limits interpretation and comparison with previously reported studies; however, the survey was developed by the multi-stakeholder iConquerMS Research Committee, comprising MS specialist health professionals and scientists, and pwMS, therefore results should be considered alongside other research for practice translation in pwMS. The non-exclusive lifestyle option selections, lack of capture of duration and adherence to behaviors, as well as researcher-defined broad re-categorizations, potentially masked associations; these and other recommendations are being considered for ongoing data capture.

## 5. Conclusions

Our study demonstrated that lifestyle behaviors concerning diet, wellness, and particularly physical activity, but not vitamin D or omega-3 intake, have positive associations with specific QoL subdomains in pwMS. Some differences in associations between RRMS and ProgMS phenotypes were observed, suggesting a need for phenotype-specific recommendations for MS management. Our findings suggest a role for modifiable lifestyle behaviors as a potential intervention for improving QoL in pwMS. Replication and validation through prospective studies are required to make specific recommendations; however, the presence and absence of associations by phenotype found in our study suggest areas that may be most rewarding for study among certain subgroups.

## Figures and Tables

**Table 1:** Lifestyle Behavior Categories.

Lifestyle	Category	Inclusions
Diet	Anti-inflammatory	Anti-inflammatory, fasting/calorie restriction, gluten-free, Mediterranean
	Low-saturated fats	Jelinek, Swank, low-fat, ovo-lactovegetarian, vegetarian, vegan, lacto-vegetarian, Ornish, Pritikin, pescatarian
	Low-carbohydrate	Atkins, ketogenic, paleo, Wahls, low-carbohydrate
	Other	Organic, low sodium, low sugar, semi-vegetarian
Supplements	Vitamin D	Vitamin D
	Omega-3	Omega-3, DHA or EPA fatty acid, fish-oil, flaxseed/flaxseed oil
Wellness	Mind	Meditation, mindfulness, guided imagery, relaxation exercise, stress management
	Mind-body	Tai chi, yoga, qigong, exercise therapy
	Other	Acupuncture, Ayurveda, biofeedback, brain training, chelation therapy, chiropractic/osteopathic manipulation, cognitive behavioral therapy, craniosacral therapy, deep breathing exercises, energy healing, hypnosis, massage, naturopathy, progressive relaxation, reflexology, traditional healing
Physical activity	Sedentary Moderate Active	leisure activity score <14 leisure activity score 14–23 leisure activity score >23

**Table 2:** (**A**). Characteristics of participants with RRMS and ProgMS. (**B**). Mean QoL T-scores of participants with RRMS and ProgMS.

(**A**)							
**Demographics**	**RRMS** ***n* = 750**	**ProgMS** ***n* = 358**	***p***	**Lifestyle Behaviors**	**RRMS** ***n* = 750**	**ProgMS** ***n* = 358**	***p***
	*n* (%)	*n* (%)			*n* (%)	*n* (%)	
Age, years (Mean, SD)	49.9 (11)	58.3 (9)	**<0.001**	**Diet**			
Sex				Anti-inflammatory			
Male	126 (17%)	113 (32%)	Ref.	Not used	464 (62%)	245 (68%)	Ref.
Female	621 (83%)	244 (68%)	**<0.001**	Used/helpful	286 (38%)	113 (32%)	**<0.05**
Country of birth				Low saturated fat			
US	634 (85%)	308 (86%)	Ref.	Not used	601 (80%)	300 (84%)	Ref.
Other	114 (15%)	50 (14%)	0.59	Used/helpful	149 (20%)	58 (16%)	0.16
Ethnicity				Low carbohydrate			
Caucasian	688 (92%)	337 (95%)	Ref.	Not used	576 (77%)	280 (78%)	Ref.
Other/mixed	56 (8%)	18 (5%)	0.15	Used/helpful	174 (23%)	78 (22%)	0.61
University degree				Other			
Yes	525 (70%)	245 (68%)	Ref.	Not used	500 (67%)	252 (70%)	Ref.
No	222 (30%)	113 (32%)	0.55	Used/helpful	250 (33%)	106 (30%)	0.23
Partnered				**Supplements**			
Yes	530 (71%)	274 (77%)	Ref.	Vitamin D			
No	214 (29%)	84 (24%)	0.06	No	108 (14%)	65 (18%)	Ref.
Paid employment				Yes	642 (86%)	293 (82%)	0.10
Yes	402 (54%)	94 (27%)	Ref.	Omega-3			
No	338 (46%)	260 (74%)	**<0.001**	Not used	461 (61%)	210 (59%)	Ref.
Household income (USD)				Used/helpful	289 (39%)	148 (41%)	0.36
≤$50,000	144 (35%)	67 (42%)	Ref.	**Wellness**			
$50,001–100,000	121 (29%)	49 (31%)	0.56	Mind			
≥$100,001	146 (35%)	44 (28%)	0.06	Not used	473 (63%)	231 (65%)	Ref.
BMI				Used/helpful	277 (37%)	127 (36%)	0.65
Under/healthy	331 (44%)	166 (47%)	Ref.	Mind-body			
Overweight	204 (27%)	103 (29%)	0.99	Not used	456 (61%)	224 (63%)	Ref.
Obese	212 (28%)	86 (24%)	0.19	Used/helpful	294 (39%)	134 (37%)	0.58
PDDS				Other			
Normal/mild	448 (60%)	36 (10%)	Ref.	Not used	249 (33%)	132 (37%)	Ref.
Moderate	252 (34%)	186 (52%)	**<0.001**	Used/helpful	501 (67%)	226 (63%)	0.24
Severe	50 (7%)	135 (38%)	**<0.001**	**Physical activity**			
MS duration (years)	11.5 (9.1)	16.1 (10.3)	**<0.001**	Sedentary	225 (30%)	154 (43%)	Ref.
				Moderate	150 (20%)	84 (24%)	0.25
				Active	375 (50%)	120 (34%)	**<0.001**
(**B**)							
**QoL Subdomains**		**RRMS** ***n* = 750**		**ProgMS** ***n* = 358**		***p***	
		Mean (SD) T-score		Mean (SD) T-score			
**Physical**							
Mobility		48.0 (9.0)		**38.30 (7.5)**		**<0.001**	
Fine motor		46.4 (8.7)		**41.0 (9.2)**		**<0.001**	
Fatigue		51.8 (9.5)		53.3 (8.5)		**<0.05**	
Sleep disturbance		53.8 (9.0)		53.4 (7.8)		0.433	
**Mental**							
Anxiety		51.8 (8.6)		50.6 (7.6)		**<0.05**	
Depression		47.7 (8.3)		48.4 (7.9)		0.185	
Positive affect		52.5 (8.2)		51.1 (8.1)		**<0.01**	
Emotional dyscontrol		48.6 (9.8)		48.1 (9.2)		0.394	
Stigma		49.0 (7.8)		52.7 (6.9)		**<0.001**	
Cognitive function		45.8 (10.7)		46.6 (10.2)		0.259	
Communication ^a^		22.1 (3.4)		21.9 (3.5)		0.390	
**Social**							
Participation		46.8 (7.9)		**43.7 (6.4)**		**<0.001**	
Satisfaction		45.3 (7.0)		**42.0 (5.5)**		**<0.001**	

BMI = body mass index; MS = multiple sclerosis; PDSS = Patient Determined Disease Steps; ProgMS = progressive MS; Ref. = reference; RRMS = relapsing-remitting MS; SD = standard deviation; USD = United States Dollar. *p* values indicate statistical differences between RRMS and ProgMS, where bolded values indicate significance (*p* < 0.05). ^a^ Total raw score. Bolded mean scores indicate differences > 5 points on the T-scale metric (0.5 SD) than the clinical or US general population.

**Table 3:** (**A**). Associations between diet and QoL subdomains, in RRMS and ProgMS. (**B**). Associations between wellness activities and QoL subdomains, in RRMS and ProgMS. (**C**). Associations between supplement use, physical activity and QoL subdomains, in RRMS and ProgMS.

(**A**)								
**Diet**	**Anti-Inflammatory**		**Low Carbohydrate**		**Low-Saturated Fat**		**Other**	
**QoL**	**RRMS**	**ProgMS**	**RRMS**	**ProgMS**	**RRMS**	**ProgMS**	**RRMS**	**ProgMS**
**Physical**								
Mobility	−0.09 (−0.97, 0.79)	**1.51 (0.17, 2.84)** *	−0.34 (−1.35, 0.67)	−0.77 (−0.73, 2.28)	0.10 (−0.97, 1.18)	0.62 (−1.09, 2.34)	−0.64 (−1.56, 0.27)	**1.42 (0.07, 2.78)** *
Fine motor	−1.06 (−2.26, 0.14)	0.69 (−1.12, 2.50)	−1.09 (−2.47, 0.28)	−0.84 (−2.87, 1.19)	0.35 (−1.11, 1.81)	1.42 (−0.90, 3.74)	**−1.24 (−2.50, −0.01)**	−0.20 (−2.05, 1.64)
Fatigue	−0.28 (−1.57, 1.00)	−0.02 (−1.96, 1.92)	0.83 (−0.64, 2.30)	−0.93 (−3.11, 1.24)	0.59 (−0.97, 2.15)	−1.53 (−4.01, 0.95)	1.02 (−0.31, 2.34)	−1.51 (−3.48, 0.47)
Sleep disturbance	0.41 (−0.81, 1.64)	0.38 (−1.47, 2.23)	−0.16 (−1.56, 1.24)	0.92 (−1.15, 2.99)	0.15 (−1.33, 1.64)	0.00 (−2.36, 2.37)	0.16 (−0.21, 2.32)	1.01 (−0.87, 2.89)
**Mental**								
Anxiety	0.08 (−1.11, 1.29)	−0.01 (−1.81, 1.80)	0.03 (−1.34, 1.40)	−0.50 (−2.53, 1.53)	0.89 (−0.56, 2.34)	1.36 (−0.94, 3.65)	0.69 (−0.55, 1.93)	0.08 (−1.76, 1.93)
Depression	0.24 (−0.94, 1.42)	−1.08 (−2.85, 0.70)	−0.11 (−1.46, 1.24)	−1.31 (−3.31, 0.68)	0.38 (−1.05, 1.81)	1.50 (−0.75, 3.76)	1.03 (−0.19, 2.25)	−0.72 (−2.53, 1.09)
Positive affect	0.45 (−0.73, 1.63)	**2.05 (0.27, 3.83)**	0.54 (−0.81, 1.88)	**2.22 (0.23, 4.21)**	0.14 (−1.29, 1.57)	−0.03 (−2.31, 2.25)	−0.17 (−1.39, 1.05)	1.55 (−0.27, 3.36)
Emotional dyscontrol	0.51 (−0.87, 1.91)	−0.08 (−2.17, 2.02)	0.20 (−1.38, 1.79)	−1.73 (−4.08, 0.62)	0.63 (−1.05, 2.32)	−0.28 (−2.97, 2.40)	0.91 (−0.53, 2.34)	−0.00 (−2.14, 2.13)
Stigma	**1.26 (0.22, 2.30)**	−0.12 (−1.68, 1.44)	**1.23 (0.04, 2.41)**	0.38 (−1.37, 2.13)	0.55 (−0.71, 1.80)	0.25 (−1.76, 2.25)	**1.36 (0.29, 2.43)**	−0.08 (−1.68, 1.52)
Cognitive function	−0.77 (−2.27, 0.72)	−0.32 (−2.58, 1.94)	−1.04 (−2.75, 0.67)	0.23 (−2.30, 2.77)	−1.04 (−2.85, 0.77)	1.64 (−1.25, 4.53)	**−1.92 (−3.46, −0.37)**	0.50 (−1.80, 2.79)
Communication ^a^	−0.20 (−0.68, 0.29)	0.07 (−0.66, 0.80)	−0.03 (−0.58, 0.52)	−0.06 (−0.88, 0.76)	−0.30 (−0.88, 0.29)	**1.23 (0.31, 2.15)** *	−0.48 (−0.98, 0.02)	0.26 (−0.48, 1.00)
**Social**								
Participation	0.27 (−0.73, 1.27)	−0.00 (−1.51, 1.51)	−0.49 (−1.63, 0.66)	−0.28 (−1.98, 1.42)	−0.53 (−1.75, 0.69)	0.38 (−1.54, 2.31)	−1.04 (−2.07, 0.01)	0.50 (−1.03, 2.04)
Satisfaction	0.28 (−0.62, 1.18)	0.77 (−0.59, 2.13)	−0.27 (−1.29, 0.76)	0.05 (−1.48, 1.57)	0.15 (−0.95, 1.24)	0.58 (−1.16, 2.33)	−0.20 (−1.13, 0.73)	0.51 (−0.87, 1.90)
(**B**)								
**Wellness**			**Mind**		**Mind−Body**		**Other**	
**QoL**			**RRMS**	**ProgMS**	**RRMS**	**ProgMS**	**RRMS**	**ProgMS**
**Physical**								
Mobility			−0.82 (−1.71, 0.07)	0.29 (−1.02, 1.61)	0.20 (−0.69, 1.09)	0.95 (−0.34, 2.24)	−0.89 (−1.80, 0.03)	0.48 (−0.81, 1.78)
Fine motor			**−1.70 (−2.90, −0.49**)	0.09 (−1.68, 1.86)	−0.34 (−1.54, 0.87)	0.57 (−1.18, 2.32)	**−1.75 (−2.99, −0.51)**	0.64 (−1.11, 2.39) *
Fatigue			**1.39 (0.10, 2.68)**	1.26 (−0.64, 3.16)	−0.83 (−2.12, 0.45)	−1.46 (−3.33, 0.41)	**2.35 (1.03, 3.68)**	0.85 (−1.03, 2.72)
Sleep disturbance			1.09 (−0.13, 2.31)	1.72 (−0.09, 3.53)	−1.16 (−2.38, 0.07)	−0.31 (−2.09, 1.47)	**1.72 (0.46, 2.99)**	0.57 (−1.22, 2.34)
**Mental**								
Anxiety			**2.30 (1.10, 3.49)**	1.57 (−0.20, 3.33)	−1.00 (−2.20, 0.20)	−0.62 (−2.36, 1.12)	**1.87 (0.63, 3.11)**	0.42 (−1.32, 2.16)
Depression			0.95 (−0.23, 2.13)	1.28 (−0.46, 3.02)	−1.06 (−2.24, 0.12)	−0.39 (−0.22, 3.20)	0.92 (−0.30, 2.14)	0.23 (−1.49, 1.95)
Positive affect			−0.40 (−1.58, 0.78)	−0.73 (−2.48, 1.02)	**1.44 (0.27, 2.62)**	1.49 (−0.22, 3.20)	−0.56 (−1.80, 0.66)	0.11 (−1.61, 1.84)
Emotional dyscontrol			**2.03 (0.64, 3.42)**	1.58 (−0.47, 3.63)	−1.63 (−3.02, −0.25)	0.50 (−1.51, 2.52)	**2.16 (0.72, 3.59)**	1.16 (−0.87, 3.17)
Stigma			**1.04 (0.01, 2.08)**	0.86 (−0.68, 2.40)	0.15 (−0.89, 1.19)	−0.40 (−1.91, 1.11)	**1.11 (0.04, 2.19)**	0.23 (−1.29, 1.75)
Cognitive function			**−1.67 (−3.16, −1.17)**	−1.38 (−3.59, 0.84)	0.76 (−0.74, 2.26)	0.26 (−1.92, 2.43)	**−3.20 (−4.74, −1.67)**	−0.89 (−3.06, 1.28)
Communication^a^			**−0.53 (−1.01, −0.05)**	−0.42 (−1.13, 0.29)	0.01 (−0.47, 0.50)	0.12 (−0.58, 0.82)	**−0.56 (−1.06, −0.07)**	−0.39 (−1.10, 0.31)
**Social**								
Participation			**−1.29(−2.29, −0.28)**	−0.89 (−2.37, 0.59)	**1.29 (0.29, 2.29)**	1.26 (−0.20, 2.71)	**−1.52 (−2.55, −0.49)**	−0.99 (−2.45, 0.47)
Satisfaction			−0.99 (−1.90, −0.09)	−0.62 (−1.95, 0.71)	0.83 (−0.08, 1.72)	1.11 (−0.20, 2.42)	**−1.24 (−2.18, −0.31)**	−0.48 (−1.80, 0.83)
(**C**)								
	**Supplements**				**Physical Activity**			
	**Vitamin D**		**Omega−3**		**Moderate**		**Active**	
**QoL**	**RRMS**	**ProgMS**	**RRMS**	**ProgMS**	**RRMS**	**ProgMS**	**RRMS**	**ProgMS**
**Physical**								
Mobility	0.01 (−1.22, 1.24)	0.40 (−1.20, 2.01)	0.35 (−0.52, 1.25)	0.33 (−0.94, 1.60)	**1.41 (0.19, 2.63)**	−0.31 (−1.88, 1.25)	**3.07 (2.04, 4.09)**	**1.49 (0.05, 2.94)**
Fine motor	−0.39 (−2.05, 1.28)	1.80 (−0.37, 3.97)	−0.34 (−1.54, 0.86)	0.76 (−0.96, 2.47)	0.93 (−0.74, 2.61)	−0.77 (−2.92, 1.38)	**2.45 (1.05, 3.86)**	−0.71 (−2.70, 1.27) *
Fatigue	−1.57 (−3.35, 0.21)	−0.92 (−3.24, 1.41)	−0.61 (−1.90, 0.67)	−0.91 (−2.75, 0.92)	−1.48 (−3.25, 0.29)	−1.35 (−3.63, 0.93)	**−4.14 (−5.63, −2.65)**	**−2.11 (−4.21, −0.01)**
Sleep disturbance	−0.52 (−2.22, 1.17)	−0.52 (−2.73, 1.70)	−0.39 (−1.61, 0.83)	−0.74 (−2.49, 1.01)	−1.20 (−2.91, 0.51)	−1.64 (−3.84, 0.55)	**−2.37 (−3.80, −0.94)**	−1.54 (−3.56, 0.48)
**Mental**								
Anxiety	0.12 (−1.53, 1.79)	−1.05 (−3.22, 1.12)	0.39 (−0.89, 1.50)	−0.48 (−2.19, 1.23)	**−1.97 (−3.64, −0.31)**	−1.66 (−3.79, 0.48)	**−2.92 (−4.32, −1.52)**	0.37 (−1.60, 2.34) *
Depression	−0.42 (−2.05, 1.22)	−0.28 (−2.41, 1.85)	0.38 (−0.80, 1.55)	0.05 (−1.63, 1.73)	**−2.02 (−3.65, −0.39)**	−1.47 (−3.56, 0.62)	**−3.67 (−5.04, −2.30)**	−0.50 (−2.43, 1.43) *
Positive affect	0.37 (−1.26, 2.01)	−0.82 (−2.95, 1.31)	−0.09 (−1.26, 1.09)	−0.05 (−1.74, 1.63)	**2.51 (0.89, 4.13)**	**2.61 (0.52, 4.69)**	**4.35 (2.99, 5.71)**	0.59 (−1.32, 2.50) *
Emotional dyscontrol	−0.28 (−2.20, 1.65)	−1.09 (−3.60, 1.42)	0.43 (−0.95, 1.82)	0.30 (−1.68, 2.28)	−1.27 (−3.41, 0.66)	−2.15 (−4.63, 0.33)	**−3.11 (−4.73, −1.49)**	0.05 (−2.33, 2.24) *
Stigma	−0.61 (−2.05, 0.83)	−1.28 (−3.15, 0.60)	0.41 (−0.61, 1.45)	−0.23 (−1.71, 1.25)	**−2.30 (−3.73, −0.86)**	−1.51 (−3.36, 0.33)	**−3.11 (−4.32, −1.90)**	−0.30 (−2.01, 1.40) *
Cognitive function	1.15 (−0.92, 3.23)	2.00 (−0.70, 4.70)	−0.07 (−1.56, 1.42)	1.39 (−0.75, 3.52)	1.49 (−0.60, 3.57)	**3.17 (0.50, 5.85)**	**2.85 (1.10, 4.60)**	1.11 (−1.37, 3.57)
Communication ^a^	0.12 (−0.56, 0.78)	0.73 (−0.14, 1.60)	−0.28 (−0.76, 0.20)	0.42 (−0.26, 1.11)	0.37 (−0.30, 1.04)	**0.93 (0.07, 1.79)**	**0.80 (0.23, 1.36)**	0.30 (−0.49, 1.10)
**Social**								
Social participation	0.69 (−0.70, 2.08)	0.82 (−1.00, 2.63)	−0.06 (−1.06, 0.94)	0.44 (−0.99, 1.87)	1.29 (−0.10, 2.70)	0.95 (−0.83, 2.74)	**2.97 (1.81, 4.14)**	0.80 (−0.84, 2.45) *
Social satisfaction	1.01 (−0.24, 2.26)	0.32 (−1.31, 1.95)	0.73 (−0.16, 1.64)	0.10 (−1.19, 1.39)	**1.64 (0.41, 2.89)**	1.49 (−0.09, 3.08)	**3.71 (2.67, 4.75)**	1.20 (−0.25, 2.66) *

ProgMS = progressive MS; QoL = quality of life; RRMS = relapsing-remitted MS; ^a^ Total raw score. Multivariate linear regression estimating adjusted regression coefficients (95% CI). Models adjusted for age, sex, BMI, education, disability, and duration since MS diagnosis. Bold values in (**A**) indicate significant (*p* < 0.05) associations between diet and QoL domains. Bold values in (**B**) indicate significant (*p* < 0.05) associations between wellness and QoL domains. Bold values in (**C**) indicate significant (*p* < 0.05) associations between physical activity and QoL subdomains. * Significant (* *p* < 0.05) difference between RRMS and ProgMS.

## Data Availability

Restrictions apply to the availability of these data. Data was obtained from Accelerated Cure Project for Multiple Sclerosis and may be requested from HS with the approval of the iConquerMS Research Committee.
